# The first case of septicemia due to nontoxigenic *Corynebacterium diphtheriae *in Poland: case report

**DOI:** 10.1186/1476-0711-4-8

**Published:** 2005-05-05

**Authors:** Aleksandra Anna Zasada, Maria Zaleska, Regina Beata Podlasin, Ilona Seferyńska

**Affiliations:** 1 Department of Bacteriology, National Institute of Hygiene, 24 Chocimska St., 00-791 Warsaw, Poland; 2 Laboratory of Microbiology, Institute of Haematology and Blood Transfusion, 5 Chocimska St., 00-957 Warsaw, Poland; 3 Hospital of Infection Disease, 37 Wolska St., 01-201 Warsaw, Poland

## Abstract

**Background:**

Toxigenic strains of *Corynebacterium diphtheriae *are well known agent of diphtheria. Nontoxigenic strains can cause atypical course of the disease. Invasive diseases caused by *C. diphtheriae *occur very rare.

**Case presentation:**

We have described the first case of septicemia and endocarditis due to nontoxigenic *C. diphtheriae *biotype *gravis *in Poland. The patient has not belonged to any group of risk such infection.

**Conclusion:**

The case presented in this article shows unusual case of infection connected with nontoxigenic *C. diphtheriae *that took place in the area where have been no cases of diphtheria and other *C. diphtheriae *infections for near ten years. It shows the importance of identifying *Corynebacterium *isolates at the species level especially when the strain has been isolated from normally sterile sites.

## Background

*Corynebacterium diphtheriae *is well known as an agent of localized respiratory tract disease potentially complicated by systemic effects of exotoxin [1,2]. It is also able to cause cutaneous and wound infections. But nontoxigenic strains can produce atypical manifestations of disease. They are able to cause diseases such as mild diphtheria-like pharyngitis, cutaneous infections, septic arthritis, abscesses, septicemia and endocarditis [3-14]. The pathogenesis of infection caused by nontoxigenic *C. diphtheriae *is unknown.

We have described the first case of bacteremia and endocarditis due to nontoxigenic *C. diphtheriae *var. *gravis *in Poland. Since 1996 there was no cases of *C. diphtheriae *infections in Poland area.

## Case presentation

In January 2004 previously healthy 38-year-old man was admitted to hospital with fever (40°C) and arthralgia of upper and lower extremities. He was not addicted to drugs or alcohol. He smoked about 20 cigarettes per day. Physical examination revealed mild tachycardia with normal cardiac sounds and normal blood pressure. Oral cavity examination showed poor dentition. He presented generalized arthralgia and edema and redness of involved joints. Also there was a 3 day history of papulomacular haemorrhagic rash on his lower extremities. The patient had no symptoms of respiratory tract infection and X-ray examination showed no evidence of pneumonia. Laboratory evaluation revealed elevated white blood cell count (17,8 × 10^3^/μl) with 34% of band neutrophils and 51% of granulocytes, decreased platelet count (29 × 10^3^/μl), slightly elevated liver enzymes (AST 52 U/L, ALT 41 U/L, LDH 589 U/L, alkaline phosphate 150 U/L), hyperbilirubinemia (2,69 mg%), elevetad creatinine concentration (1,51 mg%), mild proteinuria, leucocyturia and erythrocyturia. The initial diagnosis was septicemia. Three blood samples were drawn during two first days of hospitalization for microbiological evaluation and empirical therapy with ceftazidime and teicoplanin was started.

Nontoxigenic *C. diphtheriae *biotype *gravis *was isolated from all blood cultures. On the third day of treatment the antibiotics were changed to amikacin and ciprofloxacin according to antibiogram. Despite resolution of most symptoms and negative blood and throat swab cultures after eight days of treatment the patient was still febrile. For that reason ciprofloxacin was changed to clindamycin.

Transthoracic and transoesopharyngeal echocardiography performed on the 13th day of treatment showed two vegetations attached to the mitral and aortal valves. The patient underwent surgery for replacement of both valves. The cultures from vegetations were negative. After the operation the patient recovered. Although the cultures were negative we have supposed that the vegetations were caused by *C. diphtheriae *because the patient had no cardiac troubles before bacteremia.

Nontoxigenic *C. diphtheriae *biotype *gravis *isolated from blood cultures was identified and biotyped with use of morphological and biochemical methods as described elsewhere. Toxin production was examined *in vitro *by the conventional [1] and modified Elek test [15]. Polymerase chain reaction (PCR) was used for the detection of diphtheria toxin gene [1,16] and the PCR result was negative.

The susceptibility of isolates to 13 antibiotics was determined by the disk diffusion method accordingly to the National Committee for Clinical Laboratory Standards [17] guidelines on Mueller-Hinton II blood agar (supplemented with 5% sheep blood). However NCCLS does not define breakpoints for *Corynebacterium sp*. For that reason interpretation was done comparatively as for *Streptococcus spp*. and *Staphylococcus spp*., because some breakpoints are different for that genera. The antimicrobial disks contained penicillin, cefaclor, cefuroxime axetil, cefazolin, ceftazidime, ceftriaxone, cefepime, amikacin, meropenem, azithromycin, trimethoprim-sulfamethoxazole, vancomycin and teicoplanin. Determination of MIC (results are shown in brackets) for ampicillin (0.5 mg/L), gentamicin (0.38 mg/L), ciprofloxacin (0.125 mg/L), clindamycin (0.25 mg/L), erythromycin (0.016 mg/L), chloramphenicol (2 mg/L) and tetracycline (0.5 mg/L) was done using E-test strips. Clindamycin and erythromycin MIC breakpoints for *Streptococcus spp*. are lower than for *Staphylococcus spp*. but both interpretations showed susceptibility of examined *C. diphtheriae *strain. Ampicillin MIC breakpoints pointed to investigated strain as ampicillin resistant. The strain was also resistant to penicillin and ceftazidime and intermediate to cefuroxime axetil and ceftriaxone.

## Conclusion

*C. diphtheriae *causes systemic disease sporadically. Only 58 cases of bacteremia infections due to that microorganism were described between 1893 and 2003 [3-9]. Forty four of them were caused by nontoxigenic strains. There has been no information of bacteremia of such etiology in Poland. In previously described cases infections were connected with low socioeconomic group of people. Most of them were intravenous drug users, alcohol addicts, unemployed and homelesses [4,7-12,18,19]. Our patient does not belong to any of above mentioned risk groups. It is supposed that the predominant route for bacterial contamination and penetration into the bloodstream are various skin lesions such as cutaneous ulcers, bullous pemphigoid, scabies and open fractures [9,12,18,19]. But in the case described here no connection between bacteremia and skin lesions was found. We have supposed that mass dental caries enabled bacteria penetration into the bloodstream.

The strain isolated from the patient was resistant to penicillin and third generation cephalosporins. Penicillin G, erythromycin or amoxicillin is the reference treatment for *C. diphtheriae *infections [1,13,20] but resistance to these antibiotics was reported [1,13,14]. Our results are also in agreement with the observation of Patey et al. [7] that appreciable resistance to third generation cephalosporins exists among *C. diphtheriae *strains.

Diphtheria is still endemic in Eastern Europe and other regions of the world although it has virtually disappeared in developed countries following mass immunization in the 1940s. Current vaccine against diphtheria contains the toxoid so it protects only against the toxigenicity but not the invasiveness of *C. diphtheriae *[9]. The high rate of immunization with diphtheria toxoid may place selective pressure on the microorganism to develop other patogenicity factors. The bacterium could acquire exogenous DNA such as transposons, bacteriophages DNA or plasmids that contains single virulence genes or whole pathogenicity islands.

Nontoxigenic *C. diphtheriae *can produce atypical manifestations of disease. The pathogenesis of infection caused by nontoxigenic strains of *C. diphtheriae *is unknown and requires investigation. The organism is capable of tissue invasion and causing fulminant disease and appears to have a predilection for cardiac valvular endothelium and synovium [8]. The occurrence of joint involvement connected with bacteremia due to *C. diphtheriae *was also reported [7,8].

The case presented in this article and cases described in other papers show the importance of identifying *Corynebacterium *isolates from normally sterile sites at the species level. Determination of antimicrobial susceptibility has also fundamental role in success of treatment because resistance to some antimicrobial agents has been reported in *C. diphtheriae *[1,13,14]. It is worth to underlined that infections connected with *C. diphtheriae *can occur in immunized population.

## Competing interests

The author(s) declare that they have no competing interests.

## Authors' contributions

AAZ carried out molecular and phenotypic tests of toxigenicity and participated in identification and antimicrobial susceptibility tests and drafted the manuscript, MZ participated in identification and antimicrobial susceptibility tests and helped to draft the manuscript, RBP and IS participated in diagnosis, observation and treatment of the case and helped to draft the manuscript.

All authors read and approved the final manuscript.
